# Using machine learning algorithms to guide rehabilitation planning for home care clients

**DOI:** 10.1186/1472-6947-7-41

**Published:** 2007-12-20

**Authors:** Mu Zhu, Zhanyang Zhang, John P Hirdes, Paul Stolee

**Affiliations:** 1 Department of Statistics and Actuarial Science, University of Waterloo, Waterloo, ON, Canada; 2 Department of Health Studies and Gerontology, University of Waterloo, Waterloo, ON, Canada; 3 Homewood Research Institute, Homewood Health Centre, Guelph, ON, Canada; 4 School of Optometry, University of Waterloo, Waterloo, ON, Canada; 5 R.B.J. Schlegel – University of Waterloo Research Institute for Aging, Waterloo, ON, Canada

## Abstract

**Background:**

Targeting older clients for rehabilitation is a clinical challenge and a research priority. We investigate the potential of machine learning algorithms – Support Vector Machine (SVM) and *K*-Nearest Neighbors (KNN) – to guide rehabilitation planning for home care clients.

**Methods:**

This study is a secondary analysis of data on 24,724 longer-term clients from eight home care programs in Ontario. Data were collected with the RAI-HC assessment system, in which the Activities of Daily Living Clinical Assessment Protocol (ADLCAP) is used to identify clients with rehabilitation potential. For study purposes, a client is defined as having rehabilitation potential if there was: i) improvement in ADL functioning, or ii) discharge home. SVM and KNN results are compared with those obtained using the ADLCAP. For comparison, the machine learning algorithms use the same functional and health status indicators as the ADLCAP.

**Results:**

The KNN and SVM algorithms achieved similar substantially improved performance over the ADLCAP, although false positive and false negative rates were still fairly high (FP > .18, FN > .34 versus FP > .29, FN. > .58 for ADLCAP). Results are used to suggest potential revisions to the ADLCAP.

**Conclusion:**

Machine learning algorithms achieved superior predictions than the current protocol. Machine learning results are less readily interpretable, but can also be used to guide development of improved clinical protocols.

## Background

Targeting older clients for rehabilitation is a clinical challenge and a research priority [1]. For clients being assessed for home care services, the decision to provide rehabilitation (especially physical or occupational therapy) has major implications for the client's future quality of life and independence, as well as major resource implications. There is considerable evidence of the feasibility and effectiveness of rehabilitation in home-based settings [2-5]; there is also evidence that many home care clients who would benefit from rehabilitation services do not receive them [6].

Resource constraints will inevitably limit the provision of rehabilitation services, but gaps in service also reflect gaps and shortcomings in the management and use of available health information. More appropriate targeting of rehabilitation therapy could be achieved through more informed care planning, but rehabilitation decisions are particularly challenging. For acute care patients, diagnoses are often clearly defined. By contrast, rehabilitation patients have considerable variability even within specific diagnostic categories. Assessment of rehabilitation potential and the potential success of rehabilitation for older patients is not always straightforward, is often complicated by medical complexity and multiple co-morbidities [7,8], and requires management by multiple health professionals in multiple care settings [9]. Our program of research is aimed at understanding whether improved clinical decision-making, and ultimately improved client outcomes, could be achieved through more sophisticated use of routinely collected health assessment information.

In this paper, we are continuing to investigate the potential for machine learning algorithms to guide rehabilitation planning for home care clients. Machine learning involves computer programs that use experience gained from exploration of a dataset to improve performance or predictive ability. These techniques are now being used extensively in biomedical applications [10], for example in predicting the role of genes and proteins. There has been less use in support of clinical decision-making and prediction, but these applications are increasing [11,12]. There has been limited investigation of machine learning techniques in predicting rehabilitation outcomes [13,14]. Although some of these results have been ambiguous [14], continued exploration in rehabilitation seems warranted given the importance and challenges of predicting rehabilitation potential or outcomes [1,15,16]. Also, large databases are becoming available in rehabilitation settings, such as those based on the Functional Independence Measure (FIM™, property of Uniform Data System for Medical Rehabilitation, a division of UB Foundation Activities, Inc) or the interRAI assessment systems [17], that could be used for this purpose.

In our previous work on the prediction of rehabilitation potential, we applied a simple machine-learning algorithm known as the *K*-nearest neighbors (KNN) algorithm, which, we argued, resembles clinical logic in that predictions are based on outcomes experienced for similar patients [18]. We found that KNN made significantly better predictions than the clinical assessment protocol – the "ADLCAP" – currently in use within the health assessment information system used for home care clients in Ontario, Canada and other jurisdictions [19]. In this article, we report two follow-up studies (Study 1 and Study 2). The results and insights gained from these studies are then used to inform potential revisions to the ADLCAP, and an initial assessment of the new method is given.

## Methods

For both studies reported below, ethics approval was obtained from the Office of Research Ethics at the University of Waterloo.

### Study 1 Methods. Making predictions with support vector machines

#### Background

In our earlier paper [18], we speculated that the support vector machine (SVM, [20]) could potentially improve upon the *K*-nearest neighbors (KNN) algorithm in two ways. First, being a state-of-the-art machine-learning algorithm and a much more flexible kernel method than KNN, SVM may give more accurate predictions. Second, the decision rule from SVM will only depend on a subset of observations – called *support vectors*; these support vectors can be regarded as prototypes and, if the total number of support vectors turns out to be small, SVM will produce a much more parsimonious and interpretable model.

#### Data

We use the same data as used in our earlier report [18]: RAI-HC data from eight Ontario Community Care Access Centres (CCACs, the organizations that coordinate the provision of home care and long-term care services in the province), consisting of 24,724 clients [mean age: 76.3 (sd = 13.9); 68.9% female; 15.7% with Alzheimer disease or other dementia]. The true rehabilitation potential (*y*) of these clients can be reliably assessed from linked health service utilization data. For study purposes, a client is defined as having rehabilitation potential if there was: i) improvement in ADL functioning, or ii) discharge home. Improvement in ADL functioning was defined as any improvement in the interRAI ADL Long Form scale derived from the RAI-HC [21], over a follow-up period of approximately one year. The rationale for this definition is that for frail older clients for whom the likely course is functional decline, any improvement in ADL functioning is important. Also, persons discharged from home care who remain in their own homes (i.e., are not admitted to a long-term care home) can also be considered to have had a successful outcome. The interRAI/Minimum Data Set instruments are a comprehensive assessment and problem identification system developed by an international consortium of researchers (interRAI). The RAI-HC is mandated for use in all of Ontario's CCACs for all longer-term clients (approximately 50% of the overall CCAC case load). Repeat assessments are completed at intervals of approximately 180 days. Assessment items include: personal items, referral information, diagnoses, cognition, communication and sensory functioning, mood and behavior, physical functioning, continence, nutrition status, oral health, skin condition, environmental issues, informal support services and service utilization, and other information. Clinical Assessment Protocols (CAPs) are triggered when specified combinations of assessment items indicate that problems or risks are present that warrant intervention or further investigation [19]. The CAP most relevant to rehabilitation planning is the Activities of Daily Living Clinical Assessment Protocol, or ADLCAP.

In our earlier work we compared results using the ADLCAP with results obtained using the KNN machine learning algorithm [18]. In order to make conservative and fair comparisons with the ADLCAP, we used only covariates that are in the ADLCAP – 19 altogether. Moreover, we also interpreted these covariates in the same way as the ADLCAP. For example, the ADLCAP treats the predictor h2a (mobility in bed) in the following way:

**if **h2a = 2, 3, 4, 5, 6, or 8 (indicating levels of dependence);

**then **consider as dependent;

**else **(meaning h2a = 0 or 1, indicating independence) consider as independent.

In other words, suppose that client A has h2a = 2 and client B has h2a = 6. The ADLCAP does not distinguish these two clients with regard to h2a. Therefore, we can recode h2a as a binary variable as follows: recode 2, 3, 4, 5, 6, and 8 as one and recode all other values (0 and 1) as zero. Table 1 summarizes how the covariates are recoded according to ADLCAP. Also included in Table 1 are the percent of clients in the dataset for whom each covariate is present (% = 1), the chi-square statistic for testing the correlation between each covariate and the response, and the Pearson correlation (corr.) between each covariate and the response. (Since the response is binary and covariates here are also recoded as binary, the Pearson correlation is not exactly the right correlation coefficient to use. The chi-square statistic is more commonly used. However, the usual Pearson correlation coefficients are still included here for the following reason. Since we have a sample size of about 25,000, the chi-square statistics are all very large, reflecting the well-known caveat of classical hypothesis testing that one can reject any null hypothesis with a large enough sample size. The absolute magnitude of these chi-square statistics should not be interpreted in the usual way, but their relative magnitude is still meaningful.)

**Table 1:** Recoding and descriptive statistics for ADL covariates

**Covariates and Description**		**Original Value**	**Recoded Value**	**% = 1**	**Chi-square**	**Corr**
h2a	Mobility in bed (moving to/from lying in bed)	0, 1, 2, 3, 4, 5, 6, 8	0, 1 → 0; else 1	9.5	42.67	-0.04
h2b	Transferring (moving to/from bed or chair)	0, 1, 2, 3, 4, 5, 6, 8	0, 1 → 0; else 1	18.0	29.32	-0.03
h2c	Locomotion in home	0, 1, 2, 3, 4, 5, 6, 8	0, 1 → 0; else 1	14.8	38.56	-0.04
h2d	Locomotion outside of home	0, 1, 2, 3, 4, 5, 6, 8	0, 1 → 0; else 1	38.2	40.98	-0.04
h2e	Dressing upper body	0, 1, 2, 3, 4, 5, 6, 8	0, 1 → 0; else 1	32.0	138.18	-0.07
h2f	Dressing lower body	0, 1, 2, 3, 4, 5, 6, 8	0, 1 → 0; else 1	37.8	76.20	-0.06
h2g	Eating	0, 1, 2, 3, 4, 5, 6, 8	0, 1 → 0; else 1	10.4	87.05	-0.06
h2h	Toilet use	0, 1, 2, 3, 4, 5, 6, 8	0, 1 → 0; else 1	19.8	125.64	-0.07
h2i	Personal hygiene (grooming)	0, 1, 2, 3, 4, 5, 6, 8	0, 1 → 0; else 1	25.6	164.36	-0.08
h2j	Bathing	0, 1, 2, 3, 4, 5, 6, 8	0, 1 → 0; else 1	77.9	1488.07	-0.25
c3	Ability to understand others	0, 1, 2, 3, 4	0, 1, 2 → 0; else 1	4.7	74.93	-0.06
p6	Overall change in care need (deterioration)	0, 1, 2	0, 1 → 0; else 1	34.8	364.56	0.12
h3	ADL decline (past 90 days)	0, 1	0, 1	39.8	326.49	0.12
k8b	Conditions or diseases causing instability	0, 1	0, 1	29.1	53.34	-0.05
k8c	Flare-up of recurrent/chronic problem	0, 1	0, 1	7.8	6.23	0.02
k8d	Treatment changed in last 30 days	0, 1	0, 1	16.6	390.95	0.13
h7a	Client optimistic about functional improvement	0, 1	0, 1	22.6	1231.44	0.22
h7b	Caregivers optimistic about functional improvement	0, 1	0, 1	11.4	726.04	0.17
h7c	Good prospect of recovery from current conditions	0, 1	0, 1	10.7	1261.85	0.23

#### The Support Vector Machine

The SVM [20] is a prediction algorithm that has received a tremendous amount of attention in the machine learning community during the last decade. Suppose *x*_*new *_is a vector containing all the covariates for a new observation. To predict its outcome, SVM uses quadratic programming to construct a model of the following form:

f(xnew)=w0+∑xi∈SVwiK(xnew;xi),
 MathType@MTEF@5@5@+=feaagaart1ev2aaatCvAUfKttLearuWrP9MDH5MBPbIqV92AaeXatLxBI9gBaebbnrfifHhDYfgasaacPC6xNi=xI8qiVKYPFjYdHaVhbbf9v8qqaqFr0xc9vqFj0dXdbba91qpepeI8k8fiI+fsY=rqGqVepae9pg0db9vqaiVgFr0xfr=xfr=xc9adbaqaaeGacaGaaiaabeqaaeqabiWaaaGcbaGaemOzayMaeiikaGIaemiEaG3aaSbaaSqaaiabd6gaUjabdwgaLjabdEha3bqabaGccqGGPaqkcqGH9aqpcqWG3bWDdaWgaaWcbaGaeGimaadabeaakiabgUcaRmaaqafabaGaem4DaC3aaSbaaSqaaiabdMgaPbqabaGccqWGlbWsdaqadaqaaiabdIha4naaBaaaleaacqWGUbGBcqWGLbqzcqWG3bWDaeqaaOGaei4oaSJaemiEaG3aaSbaaSqaaiabdMgaPbqabaaakiaawIcacaGLPaaaaSqaaiabdIha4naaBaaameaacqWGPbqAaeqaaSGaeyicI4Saem4uamLaemOvayfabeqdcqGHris5aOGaeiilaWcaaa@5320@

where *w*_0 _and *w*_*i *_are model coefficients; and *K*(*u*;*v*) is a kernel function. Once the parameters *w*_0 _and *w*_*i *_are estimated, the final model depends only on a subset of the training data, denoted above by "SV" – they are called *support vectors *and are automatically determined by the SVM algorithm. To fit the SVM, we use a library called "e1071" in R [22].

#### Performance Evaluation

To fit an SVM model, we choose the default kernel function, the radial basis kernel. Among the four options provided by the "e1071" library – linear, polynomial, radial basis, and sigmoid – the radial basis kernel is also the most compatible with a distance-based method such as the KNN. To use SVM with the radial basis kernel, there are two tuning parameters that we must specify *a priori*: one that controls the width of the kernel function, which we denote here by *γ*; and another that essentially controls how many support vectors the algorithm will ultimately select, which we denote here by *C*. The performance of SVM is sensitive to these tuning parameters and the optimal value of these parameters are problem-specific.

To determine the best values of these parameters for our problem and evaluate the final predictive power of SVMs, we use the same analytic framework as in our earlier study [18]. In particular, we make predictions for the eight CCAC datasets one by one. For example, when making predictions for region 1, we randomly sample 2500 observations from regions 2–8 and use them as the training set for building the SVM. Tuning parameters are selected by performing 5-fold cross-validation on the training set alone using the overall error rate as the guiding criterion. Data from region 1 are not used to build the SVM or select the tuning parameters. This procedure guarantees that our SVMs do not use any information from the data they are about to predict, so their predictive performances can be fairly evaluated.

Since we construct and use different training sets to make predictions for different regions, we have a total of eight different training sets. After performing cross-validation on each of them, eight slightly different sets of optimal tuning parameters are obtained. They turn out to be quite close to each other. Generally, the optimal parameters are around *γ *= 0.06 and *C *= 0.24.

Prediction accuracy is then evaluated using exactly the same four criteria as in [18], namely, the false positive (false+) and false negative (false-) rates, and the positive and negative diagnostic likelihood ratio (DLR+ and DLR-).

### Study 2 Methods. Relaxation of covariates

#### Background

We have pointed out that the ADLCAP seemed to interpret the covariates in a rather restrictive manner [18]. For example, for variables h2a – h2j, the ADLCAP did *not *differentiate among the values 2, 3, 4, 5, 6 and 8. This might lead to a loss of information. We speculated that one could possibly improve the prediction accuracy of various machine-learning algorithms if no such arbitrary restrictions were imposed [18].

#### Data

We use the *original *RAI-HC datasets *without *recoding the variables according to Table 1, except that, for variables h2a – h2j, we recode the value 8 ("activity did not occur") into a 6 (total dependence) following conventional interRAI practice of combining "8s" with the most severe impairment level [23]. To distinguish the final datasets used in this study and the previous one, we shall refer to the ones here as the "relaxed datasets," because we have removed the restrictions imposed by the ADLCAP.

#### Data Analysis

We apply both the KNN and the SVM algorithms to the relaxed datasets and do so in exactly the same way as before, except the optimal tuning parameters – that is, the number *K *in KNN, and the numbers *γ *and *C *in SVM – have to be re-calibrated. Again, we do this with cross-validation. The parameters chosen are: *K *= 20, *C *= 1.25, and *γ *= 0.01.

## Results

### Study 1 Results. Making predictions with support vector machines

Contrary to our speculations [18], we find that, for this particular problem, SVM does not offer a statistically significant improvement over KNN in terms of prediction accuracy (Table 2). In addition, about 75% of the observations are selected by SVM as support vectors. Hence, there is hardly any gain in terms of parsimony or interpretability.

However, this does not mean that SVM is completely useless for our problem. In SVM, observations chosen as support vectors are either very close to or on the wrong side of the decision boundary. Non-support vectors, on the other hand, are on the correct side of the boundary and at least a certain distance away from it; they are the easy-to-classify observations in the dataset [20]. In our context, these are clients that, according to SVM, either clearly have or clearly do not have any rehabilitation potential. A careful examination of these two groups of clients, therefore, can yield additional insights.

We build an SVM with a random sample of 10,000 observations from all eight CCAC datasets and examine the resulting two groups of support vectors. In Table 3, each row shows the fraction of observations in each of these two group whose corresponding covariate is equal to 1 – recall from Table 1 that all covariates had been recoded in our study to be binary. It is evident from Table 3, that these two groups of clients are most different in terms of h2j, h7a, and h7c, which suggests that they are the most important variables for predicting rehabilitation potential.

**Table 2:** Prediction performance of various algorithms. "CAP" refers to the ADLCAP. Results for KNN are taken from [18].

	**Overall Error**			**False +**			**False -**			**DLR +**			**DLR -**		
**Region**	**CAP**	**KNN**	**SVM**	**CAP**	**KNN**	**SVM**	**CAP**	**KNN**	**SVM**	**CAP**	**KNN**	**SVM**	**CAP**	**KNN**	**SVM**

**1**	0.37	0.34	0.35	0.30	0.34	0.35	0.65	0.36	0.35	1.18	1.88	1.86	0.92	0.55	0.54
**2**	0.37	0.32	0.29	0.31	0.31	0.25	0.62	0.38	0.43	1.24	2.01	2.22	0.89	0.55	0.58
**3**	0.38	0.31	0.32	0.32	0.27	0.29	0.63	0.50	0.46	1.14	1.84	1.88	0.93	0.68	0.64
**4**	0.46	0.32	0.31	0.36	0.30	0.28	0.65	0.35	0.36	0.99	2.15	2.25	1.00	0.50	0.51
**5**	0.31	0.23	0.24	0.27	0.18	0.22	0.67	0.53	0.44	1.25	2.57	2.58	0.91	0.65	0.56
**6**	0.43	0.28	0.29	0.38	0.24	0.27	0.62	0.41	0.38	1.01	2.40	2.33	1.00	0.55	0.52
**7**	0.48	0.30	0.32	0.43	0.28	0.31	0.59	0.37	0.34	0.95	2.29	2.14	1.04	0.51	0.50
**8**	0.42	0.31	0.33	0.37	0.28	0.32	0.62	0.42	0.37	1.03	2.08	1.96	0.98	0.58	0.54
**Mean**	**0.40**	**0.30**	**0.31**	**0.34**	**0.28**	**0.29**	**0.63**	**0.42**	**0.39**	**1.10**	**2.15**	**2.15**	**0.96**	**0.57**	**0.55**

**Table 3:** Differences between clients who most clearly have and those who most clearly do not have not rehabilitation potential, according to SVM.

	**Clearly Have**	**Clearly No**	**Absolute**
**Covariates**	**Potential**	**Potential**	**Difference**
h2a = 1	0.01	0.10	0.09
h2b = 1	0.08	0.16	0.08
h2c = 1	0.04	0.15	0.11
h2d = 1	0.22	0.37	0.15
h2e = 1	0.11	0.32	0.21
h2f = 1	0.17	0.37	0.20
h2g = 1	0.01	0.12	0.12
h2h = 1	0.03	0.24	0.20
h2i = 1	0.07	0.27	0.20
h2j = 1	0.29	1.00	**0.71**
c3 = 1	1.00	0.94	0.06
p6 = 1	0.49	0.11	0.37
h3 = 1	0.49	0.12	0.37
k8b = 1	0.17	0.29	0.12
k8c = 1	0.05	0.05	0.00
k8d = 1	0.32	0.02	0.30
h7a = 1	0.65	0.00	**0.65**
h7b = 1	0.35	0.00	0.35
h7c = 1	0.46	0.00	**0.46**

We then perform a slightly different analysis to verify this result. Recall that, along our earlier analyses, we have created eight different training datasets, each consisting of 2500 observations. On each of these eight datasets, we perform stepwise variable selection on a standard logistic regression model using the Akaike Information Criterion (AIC) as the selection criterion. This is done with the functions "glm" and "stepAIC" in R [22]. We thus obtain eight slightly different subsets of selected variables. The only variables that appear in the intersection of *all eight *subsets are h2j – independent in bathing, h7a – client optimistic about functional improvement, and h7c – client rated as having good prospects of recovery.

#### Summary

Like KNN, SVM predicts rehabilitation potential better than the ADLCAP, but there is little statistical difference between KNN and SVM. Analysis using the SVM reveals that the most important variables for predicting rehabilitation potential are h2j – independent in bathing, h7a – client optimistic about functional improvement, and h7c – client rated as having good prospect of recovery.

### Study 2 Results. Relaxation of Covariates

The Study 2 results are peculiar and at first counter-intuitive. When the original scales are used, SVM performs slightly better but KNN performs slightly worse than before (Tables 4 and 5).

**Table 4:** Prediction performance of KNN, old versus new. "Old" = KNN results from [18], same as Table 2; "New" = KNN applied to the "relaxed datasets."

	**Overall Error**		**False +**		**False -**		**DLR +**		**DLR -**	
**Region**	**Old**	**New**	**Old**	**New**	**Old**	**New**	**Old**	**New**	**Old**	**New**

**1**	0.34	0.36	0.34	0.35	0.36	0.41	1.88	1.69	0.55	0.63
**2**	0.32	0.33	0.31	0.29	0.38	0.46	2.01	1.85	0.55	0.65
**3**	0.31	0.34	0.27	0.30	0.50	0.48	1.84	1.70	0.68	0.70
**4**	0.32	0.32	0.30	0.24	0.35	0.47	2.15	2.21	0.50	0.62
**5**	0.23	0.32	0.18	0.30	0.53	0.45	2.57	1.84	0.65	0.64
**6**	0.28	0.31	0.24	0.27	0.41	0.42	2.40	2.11	0.55	0.58
**7**	0.30	0.32	0.28	0.26	0.37	0.46	2.29	2.12	0.51	0.61
**8**	0.31	0.35	0.28	0.33	0.42	0.42	2.08	1.75	0.58	0.63
**Mean**	**0.30**	**0.33**	**0.28**	**0.29**	**0.42**	**0.45**	**2.15**	**1.91**	**0.57**	**0.63**

**Table 5:** Prediction performance of SVM, old versus new. "Old" = SVM applied to the datasets used in (18), same as Table 2; "New" = SVM applied to the "relaxed datasets."

	**Overall Error**		**False +**		**False -**		**DLR +**		**DLR -**	
**Region**	**Old**	**New**	**Old**	**New**	**Old**	**New**	**Old**	**New**	**Old**	**New**

**1**	0.35	0.34	0.35	0.34	0.35	0.33	1.86	1.97	0.54	0.50
**2**	0.29	0.30	0.25	0.26	0.43	0.42	2.22	2.20	0.58	0.57
**3**	0.32	0.34	0.29	0.33	0.46	0.42	1.88	1.77	0.64	0.63
**4**	0.31	0.31	0.28	0.28	0.36	0.37	2.25	2.24	0.51	0.51
**5**	0.24	0.23	0.22	0.20	0.44	0.45	2.58	2.69	0.56	0.57
**6**	0.29	0.29	0.27	0.26	0.38	0.40	2.33	2.36	0.52	0.53
**7**	0.32	0.31	0.31	0.29	0.34	0.35	2.14	2.23	0.50	0.49
**8**	0.33	0.32	0.32	0.30	0.37	0.38	1.96	2.04	0.54	0.55
**Mean**	**0.31**	**0.30**	**0.29**	**0.28**	**0.39**	**0.39**	**2.15**	**2.19**	**0.55**	**0.54**

In order to understand this peculiar behavior, a series of in-depth exploratory analyses are performed on the datasets. The analysis that provides us with an insight into this peculiarity is described below. The insight gained from this analysis not only resolves this mystery for us; it also suggests a new method of defining the ADLCAP.

Take the covariate h2a for example. Using all the data, we can estimate the following ratio:

rh2aj=Pr⁡(h2a=j|y=1)Pr⁡(h2a=j|y=0),j=0,1,2,⋯,6.
 MathType@MTEF@5@5@+=feaagaart1ev2aaatCvAUfKttLearuWrP9MDH5MBPbIqV92AaeXatLxBI9gBaebbnrfifHhDYfgasaacPC6xNi=xI8qiVKYPFjYdHaVhbbf9v8qqaqFr0xc9vqFj0dXdbba91qpepeI8k8fiI+fsY=rqGqVepae9pg0db9vqaiVgFr0xfr=xfr=xc9adbaqaaeGacaGaaiaabeqaaeqabiWaaaGcbaqbaeqabeGaaaqaaiabdkhaYnaaDaaaleaacqWGObaAcqaIYaGmcqWGHbqyaeaacqWGQbGAaaGccqGH9aqpjuaGdaWcaaqaaiGbccfaqjabckhaYjabcIcaOiabdIgaOjabikdaYiabdggaHjabg2da9iabdQgaQjabcYha8jabdMha5jabg2da9iabigdaXiabcMcaPaqaaiGbccfaqjabckhaYjabcIcaOiabdIgaOjabikdaYiabdggaHjabg2da9iabdQgaQjabcYha8jabdMha5jabg2da9iabicdaWiabcMcaPaaakiabcYcaSaqaaiabdQgaQjabg2da9iabicdaWiabcYcaSiabigdaXiabcYcaSiabikdaYiabcYcaSiabl+UimjabcYcaSiabiAda2iabc6caUaaaaaa@6034@

If rh2a0
 MathType@MTEF@5@5@+=feaagaart1ev2aaatCvAUfKttLearuWrP9MDH5MBPbIqV92AaeXatLxBI9gBaebbnrfifHhDYfgasaacPC6xNi=xH8viVGI8Gi=hEeeu0xXdbba9frFj0xb9qqpG0dXdb9aspeI8k8fiI+fsY=rqGqVepae9pg0db9vqaiVgFr0xfr=xfr=xc9adbaqaaeGacaGaaiaabeqaaeqabiWaaaGcbaGaemOCai3aa0baaSqaaiabdIgaOjabikdaYiabdggaHbqaaiabicdaWaaaaaa@31F1@ > 1, this means it is more likely for those with rehabilitation potential to score a zero on item h2a. Likewise, if rh2a0
 MathType@MTEF@5@5@+=feaagaart1ev2aaatCvAUfKttLearuWrP9MDH5MBPbIqV92AaeXatLxBI9gBaebbnrfifHhDYfgasaacPC6xNi=xH8viVGI8Gi=hEeeu0xXdbba9frFj0xb9qqpG0dXdb9aspeI8k8fiI+fsY=rqGqVepae9pg0db9vqaiVgFr0xfr=xfr=xc9adbaqaaeGacaGaaiaabeqaaeqabiWaaaGcbaGaemOCai3aa0baaSqaaiabdIgaOjabikdaYiabdggaHbqaaiabicdaWaaaaaa@31F1@ < 1, it means it is more likely for those without rehabilitation potential to score a zero on this item.

Call {rh2a0,rh2a1,⋯,rh2a6}
 MathType@MTEF@5@5@+=feaagaart1ev2aaatCvAUfKttLearuWrP9MDH5MBPbIqV92AaeXatLxBI9gBaebbnrfifHhDYfgasaacPC6xNi=xH8viVGI8Gi=hEeeu0xXdbba9frFj0xb9qqpG0dXdb9aspeI8k8fiI+fsY=rqGqVepae9pg0db9vqaiVgFr0xfr=xfr=xc9adbaqaaeGacaGaaiaabeqaaeqabiWaaaGcbaWaaiWabeaacqWGYbGCdaqhaaWcbaGaemiAaGMaeGOmaiJaemyyaegabaGaeGimaadaaOGaeiilaWIaemOCai3aa0baaSqaaiabdIgaOjabikdaYiabdggaHbqaaiabigdaXaaakiabcYcaSiabl+UimjabcYcaSiabdkhaYnaaDaaaleaacqWGObaAcqaIYaGmcqWGHbqyaeaacqaI2aGnaaaakiaawUhacaGL9baaaaa@4519@ the (likelihood) *ratio profile *of h2a. For the sake of argument, suppose the ratio profile of h2a looks like this: {5, 4, 3, 2, 0.5, 0.2, 0.1}. Such a profile would mean that clients *with *rehabilitation potential are 5 times more likely than those without potential to score a 0 on item h2a, 4 times more likely to score a 1, 3 times more likely to score a 2, and 2 times more likely to score a 3. On the other hand, clients *without *rehabilitation potential are 10 times more likely to score a 6, 5 times more likely to score a 5 and 2 times more likely to score a 4.

Based on such a profile, how would one use h2a (alone) to predict rehabilitation potential *y *? The obvious answer is as follows:

y^={1,if h2a=0,1,2,3,0,if h2a=4,5,6;
 MathType@MTEF@5@5@+=feaagaart1ev2aaatCvAUfKttLearuWrP9MDH5MBPbIqV92AaeXatLxBI9gBaebbnrfifHhDYfgasaacPC6xNi=xI8qiVKYPFjYdHaVhbbf9v8qqaqFr0xc9vqFj0dXdbba91qpepeI8k8fiI+fsY=rqGqVepae9pg0db9vqaiVgFr0xfr=xfr=xc9adbaqaaeGacaGaaiaabeqaaeqabiWaaaGcbaGafmyEaKNbaKaacqGH9aqpdaGabeqaauaabaqaciaaaeaacqaIXaqmcqGGSaalaeaacqqGPbqAcqqGMbGzcqqGGaaicqWGObaAcqaIYaGmcqWGHbqycqGH9aqpcqaIWaamcqGGSaalcqaIXaqmcqGGSaalcqaIYaGmcqGGSaalcqaIZaWmcqGGSaalaeaacqaIWaamcqGGSaalaeaacqqGPbqAcqqGMbGzcqqGGaaicqWGObaAcqaIYaGmcqWGHbqycqGH9aqpcqaI0aancqGGSaalcqaI1aqncqGGSaalcqaI2aGncqGG7aWoaaaacaGL7baaaaa@5082@

or

y^={1,for j such that rh2aj>1,0,for j such that rh2aj<1.
 MathType@MTEF@5@5@+=feaafiart1ev1aaatCvAUfKttLearuWrP9MDH5MBPbIqV92AaeXatLxBI9gBaebbnrfifHhDYfgasaacPC6xNi=xI8qiVKYPFjYdHaVhbbf9v8qqaqFr0xc9vqFj0dXdbba91qpepeI8k8fiI+fsY=rqGqVepae9pg0db9vqaiVgFr0xfr=xfr=xc9adbaqaaeGacaGaaiaabeqaaeqabiWaaaGcbaGafmyEaKNbaKaacqGH9aqpdaGabeqaauaabaqaciaaaeaacqaIXaqmcqGGSaalaeaacqqGMbGzcqqGVbWBcqqGYbGCcqqGGaaicqWGQbGAcqqGGaaicqqGZbWCcqqG1bqDcqqGJbWycqqGObaAcqqGGaaicqqG0baDcqqGObaAcqqGHbqycqqG0baDcqqGGaaicqWGYbGCdaqhaaWcbaGaemiAaGMaeGOmaiJaemyyaegabaGaemOAaOgaaOGaeyOpa4JaeGymaeJaeiilaWcabaGaeGimaaJaeiilaWcabaGaeeOzayMaee4Ba8MaeeOCaiNaeeiiaaIaemOAaOMaeeiiaaIaee4CamNaeeyDauNaee4yamMaeeiAaGMaeeiiaaIaeeiDaqNaeeiAaGMaeeyyaeMaeeiDaqNaeeiiaaIaemOCai3aa0baaSqaaiabdIgaOjabikdaYiabdggaHbqaaiabdQgaQbaakiabgYda8iabigdaXiabc6caUaaaaiaawUhaaaaa@6D90@

Now, define the *ratio profile score *for h2a as

sh2a=max⁡j{rh2aj:rh2aj≥1}/max⁡j{rh2aj:rh2aj<1}.
 MathType@MTEF@5@5@+=feaagaart1ev2aaatCvAUfKttLearuWrP9MDH5MBPbIqV92AaeXatLxBI9gBaebbnrfifHhDYfgasaacPC6xNi=xI8qiVKYPFjYdHaVhbbf9v8qqaqFr0xc9vqFj0dXdbba91qpepeI8k8fiI+fsY=rqGqVepae9pg0db9vqaiVgFr0xfr=xfr=xc9adbaqaaeGacaGaaiaabeqaaeqabiWaaaGcbaWaaSGbaeaacqWGZbWCdaWgaaWcbaGaemiAaGMaeGOmaiJaemyyaegabeaakiabg2da9maaxababaGagiyBa0MaeiyyaeMaeiiEaGhaleaacqWGQbGAaeqaaOWaaiWabeaacqWGYbGCdaqhaaWcbaGaemiAaGMaeGOmaiJaemyyaegabaGaemOAaOgaaOGaeiOoaOJaemOCai3aa0baaSqaaiabdIgaOjabikdaYiabdggaHbqaaiabdQgaQbaakiabgwMiZkabigdaXaGaay5Eaiaaw2haaaqaamaaxababaGagiyBa0MaeiyyaeMaeiiEaGhaleaacqWGQbGAaeqaaaaakmaacmqabaGaemOCai3aa0baaSqaaiabdIgaOjabikdaYiabdggaHbqaaiabdQgaQbaakiabcQda6iabdkhaYnaaDaaaleaacqWGObaAcqaIYaGmcqWGHbqyaeaacqWGQbGAaaGccqGH8aapcqaIXaqmaiaawUhacaGL9baacqGGUaGlaaa@644A@

In the hypothetical illustration above, we would have *S*_*h*2*a *_= 5 ÷ (0.5) = 10. This score can be treated as a rough measure of how accurately one can predict rehabilitation potential using the covariate h2a. The higher the score, the better.

**Figure 1:**
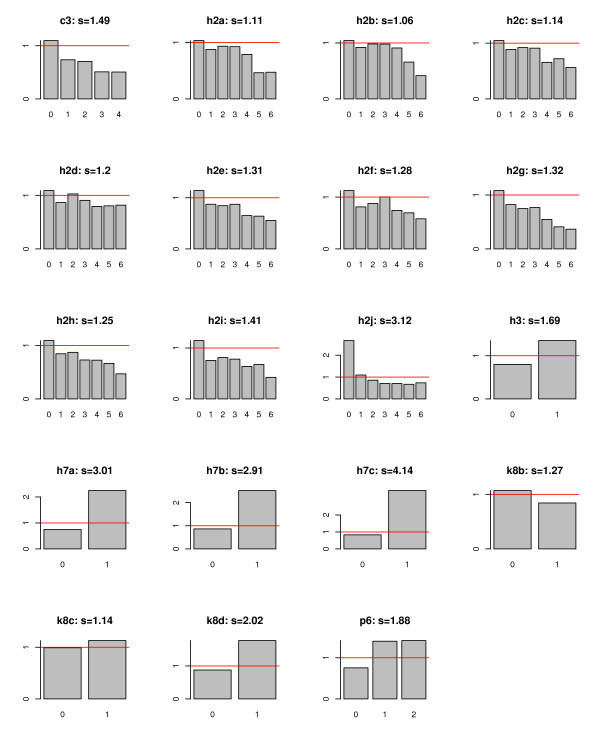
Ratio profiles for all 19 covariates, together with their ratio profile scores.

Figure 1 shows the ratio profiles of all 19 covariates together with their corresponding ratio profile scores; the horizontal line in each profile plot is the critical line at which the ratio is equal to 1. We can make the following observations:

1. The three covariates with the highest ratio profile scores are: h7c – good prospect of recovery (*S*_*h*7*c *_= 4.14), h2j – bathing (*S*_*h*2*j *_= 3.12), and h7a – client optimistic about functional improvement (*S*_*h*7*a *_= 3.01). This is, again, in exact agreement with our results from Study 1.

2. The covariates h7c and h7a are not affected whether we use the original scale or not (see Figure 1).

3. Based on the ratio profile of h2j, the best way to use h2j for prediction is as follows: y^
 MathType@MTEF@5@5@+=feaagaart1ev2aaatCvAUfKttLearuWrP9MDH5MBPbIqV92AaeXatLxBI9gBaebbnrfifHhDYfgasaacPC6xNi=xH8viVGI8Gi=hEeeu0xXdbba9frFj0xb9qqpG0dXdb9aspeI8k8fiI+fsY=rqGqVepae9pg0db9vqaiVgFr0xfr=xfr=xc9adbaqaaeGacaGaaiaabeqaaeqabiWaaaGcbaGafmyEaKNbaKaaaaa@2D5E@ = 1 if *h*2 *j *= 0, 1; y^
 MathType@MTEF@5@5@+=feaagaart1ev2aaatCvAUfKttLearuWrP9MDH5MBPbIqV92AaeXatLxBI9gBaebbnrfifHhDYfgasaacPC6xNi=xH8viVGI8Gi=hEeeu0xXdbba9frFj0xb9qqpG0dXdb9aspeI8k8fiI+fsY=rqGqVepae9pg0db9vqaiVgFr0xfr=xfr=xc9adbaqaaeGacaGaaiaabeqaaeqabiWaaaGcbaGafmyEaKNbaKaaaaa@2D5E@ = 0 if *h*2 *j *= 2, 3, 4, 5, 6. That is, for h2j, the recoded scale is actually the best, better than the original scale which uses more information on levels of impairment. Using the original scale turns out to only add extra noise to our underlying prediction problem.

4. For covariates in the h2* category (h2a – h2j), h2i has the second highest score (*S*_*h*2*i *_= 1.41). Based on its ratio profile, the best way to use h2i for prediction is as follows: y^
 MathType@MTEF@5@5@+=feaagaart1ev2aaatCvAUfKttLearuWrP9MDH5MBPbIqV92AaeXatLxBI9gBaebbnrfifHhDYfgasaacPC6xNi=xH8viVGI8Gi=hEeeu0xXdbba9frFj0xb9qqpG0dXdb9aspeI8k8fiI+fsY=rqGqVepae9pg0db9vqaiVgFr0xfr=xfr=xc9adbaqaaeGacaGaaiaabeqaaeqabiWaaaGcbaGafmyEaKNbaKaaaaa@2D5E@ = 1 if *h*2*i *= 0 ; y^
 MathType@MTEF@5@5@+=feaagaart1ev2aaatCvAUfKttLearuWrP9MDH5MBPbIqV92AaeXatLxBI9gBaebbnrfifHhDYfgasaacPC6xNi=xH8viVGI8Gi=hEeeu0xXdbba9frFj0xb9qqpG0dXdb9aspeI8k8fiI+fsY=rqGqVepae9pg0db9vqaiVgFr0xfr=xfr=xc9adbaqaaeGacaGaaiaabeqaaeqabiWaaaGcbaGafmyEaKNbaKaaaaa@2D5E@ = 0 if *h*2*i *= 1, 2, 3, 4, 5, 6. That is, for h2i, the recoded scale is *almost *the best. It would have been better to separate 0 from 1–6 rather than grouping 0 and 1 together (see Figure 1). In fact, the same can be said about most other covariates in the h2* category – except h2d. For these covariates, using the original scale adds some extra noise, but it also introduces the opportunity for an algorithm to use these covariates in a more optimal way.

5. The ratio profiles of c3 and p6 indicate that the recoded scales severely mask the information contained in these covariates for predicting rehabilitation potential. There is extra information in the original scale. In both cases, it would have been better to recode "0" into "0" and everything else into "1".

These observations suggest that there is a tradeoff between using the original and the recoded scales. On the one hand, there is some extra information useful for prediction if the original scales are used. On the other hand, the recoded scales are optimal for the most influential covariates and there is a considerable amount of added noise if the original scales are used.

Notice that our definition of the ratio profile score here is not general. Generally speaking, it would have been better to define the ratio profile score (for covariate *x*) as

s˜x=min⁡j{rxj:rxj≥1}/max⁡j{rxj:rxj<1}.
 MathType@MTEF@5@5@+=feaagaart1ev2aaatCvAUfKttLearuWrP9MDH5MBPbIqV92AaeXatLxBI9gBaebbnrfifHhDYfgasaacPC6xNi=xI8qiVKYPFjYdHaVhbbf9v8qqaqFr0xc9vqFj0dXdbba91qpepeI8k8fiI+fsY=rqGqVepae9pg0db9vqaiVgFr0xfr=xfr=xc9adbaqaaeGacaGaaiaabeqaaeqabiWaaaGcbaWaaSGbaeaacuWGZbWCgaacamaaBaaaleaacqWG4baEaeqaaOGaeyypa0ZaaCbeaeaacyGGTbqBcqGGPbqAcqGGUbGBaSqaaiabdQgaQbqabaGcdaGadeqaaiabdkhaYnaaDaaaleaacqWG4baEaeaacqWGQbGAaaGccqGG6aGocqWGYbGCdaqhaaWcbaGaemiEaGhabaGaemOAaOgaaOGaeyyzImRaeGymaedacaGL7bGaayzFaaaabaWaaCbeaeaacyGGTbqBcqGGHbqycqGG4baEaSqaaiabdQgaQbqabaaaaOWaaiWabeaacqWGYbGCdaqhaaWcbaGaemiEaGhabaGaemOAaOgaaOGaeiOoaOJaemOCai3aa0baaSqaaiabdIha4bqaaiabdQgaQbaakiabgYda8iabigdaXaGaay5Eaiaaw2haaiabc6caUaaa@59C4@

However, it can easily be seen from Figure 1 that, for most covariates in our dataset, there is only one ratio above the critical threshold of one. For these covariates, the two versions of the ratio profile scores are identical, that is, s˜x
 MathType@MTEF@5@5@+=feaagaart1ev2aaatCvAUfKttLearuWrP9MDH5MBPbIqV92AaeXatLxBI9gBaebbnrfifHhDYfgasaacPC6xNi=xH8viVGI8Gi=hEeeu0xXdbba9frFj0xb9qqpG0dXdb9aspeI8k8fiI+fsY=rqGqVepae9pg0db9vqaiVgFr0xfr=xfr=xc9adbaqaaeGacaGaaiaabeqaaeqabiWaaaGcbaGafm4CamNbaGaadaWgaaWcbaGaemiEaGhabeaaaaa@2EF6@ = *S*_*x*_. The only exceptions are: h2d and h2j. In the case of h2d, Figure 1 shows that the two bars above the critical threshold are of similar heights, i.e., min⁡j{rh2dj:rh2dj≥1}≈max⁡j{rh2dj:rh2dj≥1}
 MathType@MTEF@5@5@+=feaagaart1ev2aaatCvAUfKttLearuWrP9MDH5MBPbIqV92AaeXatLxBI9gBaebbnrfifHhDYfgasaacPC6xNi=xH8viVGI8Gi=hEeeu0xXdbba9frFj0xb9qqpG0dXdb9aspeI8k8fiI+fsY=rqGqVepae9pg0db9vqaiVgFr0xfr=xfr=xc9adbaqaaeGacaGaaiaabeqaaeqabiWaaaGcbaWaaCbeaeaacyGGTbqBcqGGPbqAcqGGUbGBaSqaaiabdQgaQbqabaGcdaGadeqaaiabdkhaYnaaDaaaleaacqWGObaAcqaIYaGmcqWGKbazaeaacqWGQbGAaaGccqGG6aGocqWGYbGCdaqhaaWcbaGaemiAaGMaeGOmaiJaemizaqgabaGaemOAaOgaaOGaeyyzImRaeGymaedacaGL7bGaayzFaaGaeyisIS7aaCbeaeaacyGGTbqBcqGGHbqycqGG4baEaSqaaiabdQgaQbqabaGcdaGadeqaaiabdkhaYnaaDaaaleaacqWGObaAcqaIYaGmcqWGKbazaeaacqWGQbGAaaGccqGG6aGocqWGYbGCdaqhaaWcbaGaemiAaGMaeGOmaiJaemizaqgabaGaemOAaOgaaOGaeyyzImRaeGymaedacaGL7bGaayzFaaaaaa@5F48@. So we expect s˜h2d
 MathType@MTEF@5@5@+=feaagaart1ev2aaatCvAUfKttLearuWrP9MDH5MBPbIqV92AaeXatLxBI9gBaebbnrfifHhDYfgasaacPC6xNi=xH8viVGI8Gi=hEeeu0xXdbba9frFj0xb9qqpG0dXdb9aspeI8k8fiI+fsY=rqGqVepae9pg0db9vqaiVgFr0xfr=xfr=xc9adbaqaaeGacaGaaiaabeqaaeqabiWaaaGcbaGafm4CamNbaGaadaWgaaWcbaGaemiAaGMaeGOmaiJaemizaqgabeaaaaa@3119@ to be very close to *S*_*h*2*d *_as well, and it does not matter very much which one is used in practice. For h2j, however, it is clear from Figure 1 that min⁡j{rh2jj:rh2jj≥1}
 MathType@MTEF@5@5@+=feaagaart1ev2aaatCvAUfKttLearuWrP9MDH5MBPbIqV92AaeXatLxBI9gBaebbnrfifHhDYfgasaacPC6xNi=xH8viVGI8Gi=hEeeu0xXdbba9frFj0xb9qqpG0dXdb9aspeI8k8fiI+fsY=rqGqVepae9pg0db9vqaiVgFr0xfr=xfr=xc9adbaqaaeGacaGaaiaabeqaaeqabiWaaaGcbaWaaCbeaeaacyGGTbqBcqGGPbqAcqGGUbGBaSqaaiabdQgaQbqabaGcdaGadeqaaiabdkhaYnaaDaaaleaacqWGObaAcqaIYaGmcqWGQbGAaeaacqWGQbGAaaGccqGG6aGocqWGYbGCdaqhaaWcbaGaemiAaGMaeGOmaiJaemOAaOgabaGaemOAaOgaaOGaeyyzImRaeGymaedacaGL7bGaayzFaaaaaa@44CB@ is much smaller than max⁡j{rh2jj:rh2jj≥1}
 MathType@MTEF@5@5@+=feaafiart1ev1aaatCvAUfKttLearuWrP9MDH5MBPbIqV92AaeXatLxBI9gBaebbnrfifHhDYfgasaacPC6xNi=xH8viVGI8Gi=hEeeu0xXdbba9frFj0xb9qqpG0dXdb9aspeI8k8fiI+fsY=rqGqVepae9pg0db9vqaiVgFr0xfr=xfr=xc9adbaqaaeGacaGaaiaabeqaaeqabiWaaaGcbaWaaCbeaeaacyGGTbqBcqGGHbqycqGG4baEaSqaaiabdQgaQbqabaGcdaGadeqaaiabdkhaYnaaDaaaleaacqWGObaAcqaIYaGmcqWGQbGAaeaacqWGQbGAaaGccqGG6aGocqWGYbGCdaqhaaWcbaGaemiAaGMaeGOmaiJaemOAaOgabaGaemOAaOgaaOGaeyyzImRaeGymaedacaGL7bGaayzFaaaaaa@44CF@, which means h2j would have had a much lower score had we used the more general definition, s˜h2j
 MathType@MTEF@5@5@+=feaagaart1ev2aaatCvAUfKttLearuWrP9MDH5MBPbIqV92AaeXatLxBI9gBaebbnrfifHhDYfgasaacPC6xNi=xH8viVGI8Gi=hEeeu0xXdbba9frFj0xb9qqpG0dXdb9aspeI8k8fiI+fsY=rqGqVepae9pg0db9vqaiVgFr0xfr=xfr=xc9adbaqaaeGacaGaaiaabeqaaeqabiWaaaGcbaGafm4CamNbaGaadaWgaaWcbaGaemiAaGMaeGOmaiJaemOAaOgabeaaaaa@3125@. But it is also clear from Figure 1 that h2j is actually one of the stronger predictive variables, and using the more general definition would have severely understated its true predictive power. Based on these considerations, we chose to use a definition that is not completely general but more suitable for our specific purposes here.

To explain the peculiar and counter-intuitive results in Tables 4 and 5, we conjecture that KNN has suffered more than benefited from this particular tradeoff, whereas SVM, being a more sophisticated and robust algorithm, has benefited more than suffered from it. This conjecture is confirmed by simulation experiments, which we describe in the Appendix.

#### Summary and new method of defining ADLCAP

The peculiar results from applying KNN and SVM to the "relaxed datasets" have led us to carry out an in-depth investigation. As a result, we are able to gain significant new insight into the nature of the problem. We find that the implicit recoding of the covariates by the ADLCAP (Table 1) is, generally speaking, quite reasonable; it is close to being optimal for the most influential covariates. More importantly, however, our investigation suggests a new method of defining the ADLCAP, one that is based on an analysis of the covariates' (likelihood) ratio profiles (Figure 1).

Figure 1 contains rich information. Take the covariate c3 for an example. The bar at c3 = 0 is higher than the critical horizontal line with height 1. This means that, if c3 = 0, it is more likely that the client has rehabilitation potential. On the other hand, the bars at c3 = 1, 2, 3, 4 are all lower than the critical line. This means if c3 = 1, 2, 3, 4, it is more likely that the client does not have rehabilitation potential. Clearly, such information can be used to make predictions. It is also clear from Figure 1 that the information contained in the ratio profile of c3 is not as good as that contained in the ratio profile of, say h7c, because h7c has a much higher ratio profile score (4.14 versus 1.49). Thus, decisions based on different covariates should be weighted accordingly. An alternative ADLCAP based on this argument is outlined in Table 6.

**Table 6:** An in-depth analysis of the covariates' (likelihood) ratio profiles (Figure 1) suggests a way to redefine the ADLCAP.

**initialize **score = 0; threshold = 15.8	
**if **(c3 = 0)	**then **score = score + 1.5;
**if **(h2a = 0)	**then **score = score + 1.1;
**if **(h2b = 0)	**then **score = score + 1.1;
**if **(h2c = 0)	**then **score = score + 1.1;
**if **(h2d = 0)	**then **score = score + 1.2;
**if **(h2e = 0)	**then **score = score + 1.3;
**if **(h2f = 0)	**then **score = score + 1.3;
**if **(h2g = 0)	**then **score = score + 1.3;
**if **(h2h = 0)	**then **score = score + 1.3;
**if **(h2i = 0)	**then **score = score + 1.4;
**if **(h2j = 0)	**then **score = score + 3.1;
**if **(h3 = 1)	**then **score = score + 1.7;
**if **(h7a = 1)	**then **score = score + 3.0;
**if **(h7b = 1)	**then **score = score + 2.9;
**if **(h7c = 1)	**then **score = score + 4.1;
**if **(k8b = 0)	**then **score = score + 1.3;
**if **(k8c = 1)	**then **score = score + 1.1;
**if **(k8d = 1)	**then **score = score + 2.0;
**if **(p6 = 1 or 2)	**then **score = score + 1.9;

**if **score > threshold;

**then **(alternative ADLCAP) = 1;

**else **(alternative ADLCAP) = 0.

The use of *threshold *= 15.8 in Table 6 is somewhat arbitrary; it is only selected so that we can make an initial assessment. On the eight CCAC datasets (*n *= 24,724), the ADLCAP is triggered for 8,913 clients, i.e., about 36.05%. To allow us to make a fair comparison, we reason backwards by asking: what would the threshold have to be so that 36.05% of all the clients would score above this threshold value on the alternative ADLCAP as well? The answer turns out to be 15.8. Table 7 shows that the predictive performance of this alternative ADLCAP is encouraging.

**Table 7:** Comparison of prediction performances. "OLD" = the original ADLCAP, same as [18] and Table 2; "SVM" = SVM using relaxed dataset, same as Table 5, column "New"; "NEW" = alternative ADLCAP (Table 6).

	**Overall Error**			**False +**			**False -**			**DLR +**			**DLR -**		
**Region**	**OLD**	**SVM**	**NEW**	**OLD**	**SVM**	**NEW**	**OLD**	**SVM**	**NEW**	**OLD**	**SVM**	**NEW**	**OLD**	**SVM**	**NEW**

**1**	0.37	0.34	0.36	0.30	0.34	0.36	0.65	0.33	0.37	1.18	1.97	1.75	0.92	0.50	0.58
**2**	0.37	0.30	0.30	0.31	0.26	0.25	0.62	0.42	0.48	1.24	2.20	2.10	0.89	0.57	0.64
**3**	0.38	0.34	0.32	0.32	0.33	0.28	0.63	0.42	0.50	1.14	1.77	1.79	0.93	0.63	0.69
**4**	0.46	0.31	0.34	0.36	0.28	0.31	0.65	0.37	0.39	0.99	2.24	1.99	1.00	0.51	0.56
**5**	0.31	0.23	0.20	0.27	0.20	0.16	0.67	0.45	0.52	1.25	2.69	3.05	0.91	0.57	0.62
**6**	0.43	0.29	0.27	0.38	0.26	0.22	0.62	0.40	0.45	1.01	2.36	2.53	1.00	0.53	0.58
**7**	0.48	0.31	0.32	0.43	0.29	0.29	0.59	0.35	0.38	0.95	2.23	2.17	1.04	0.49	0.53
**8**	0.42	0.32	0.29	0.37	0.30	0.23	0.62	0.38	0.49	1.03	2.04	2.16	0.98	0.55	0.64
**Mean**	**0.40**	**0.30**	**0.30**	**0.34**	**0.28**	**0.26**	**0.63**	**0.39**	**0.45**	**1.10**	**2.19**	**2.19**	**0.96**	**0.54**	**0.61**

## Discussion

Clients requiring rehabilitation are at a critical turning point in terms of their future functioning and quality of life, and their potential to live independently. If information systems are used to ensure appropriate and equitable access to rehabilitation services, there will be major benefits to the health, quality of life and independence of rehabilitation clients. There will also be major health system benefits through decreased costs, more appropriate resource use, and avoided institutional placements.

In the first study reported here, we found that the support vector machine (SVM) predicts rehabilitation potential better than the ADLCAP, but there is little statistical difference between SVM and the *K*-nearest neighbors (KNN) algorithm [18]. In addition, the SVM did not really give a more parsimonious model. Using the SVM, however, we were able to find that the most important predictors for this particular prediction task are dependence in bathing (h2j), the client being optimistic about functional improvement (h7a), and good prospects of recovery from current conditions (h7c). In the second study, we found that the implicit recoding of the covariates by the ADLCAP (Table 1) is generally quite reasonable, especially for the most important predictors. We then described a simple analysis based on the covariates' (likelihood) ratio profiles and showed that such an analysis can lead to a new method of defining the ADLCAP. Our initial assessment showed that the alternative ADLCAP thus defined is capable of producing predictions that are competitive against the machine learning algorithms we have experimented with so far.

We believe our work to date supports continued investigation of the potential for advanced statistical techniques, including machine learning algorithms, to support care planning for rehabilitation. Both of the machine learning techniques we have explored, the KNN and SVM algorithms, have achieved substantially improved performance over a currently used clinical protocol. Reservations about the use of these methods include the interpretability of their results, and the resulting potential for clinical resistance to a "black box" approach. For this reason we have so far chosen methods that could be seen as analogous to clinical reasoning (KNN) or that could identify prototypical cases that could aid interpretation (SVM). In addition to improved statistical prediction, our work points to an additional, and possibly more important, benefit of these methods. Our analyses of the machine learning results have provided insights into the factors that may be most influential in predicting rehabilitation potential – "the contents of the black box" – and also into optimal ways to categorize these variables (i.e., to define clinical cutpoints). We have also shown how these results could be used to redefine the clinical protocol to achieve results similar to that achieved using machine learning algorithms.

## Conclusion

Machine learning algorithms achieved superior predictions than the current protocol, although the results are less readily interpretable. We recognize that targeting clients for rehabilitation remains a challenge, and any manageable health information system will be limited in its ability to predict rehabilitation potential. We suggest however that we have illustrated how machine learning techniques can "set the bar" for clinical predictions, and also how machine learning can be used to refine clinical protocols to achieve comparable performance.

## Competing interests

The author(s) declare that they have no competing interests.

## Authors' contributions

MZ, JPH and PS all participated in the conceptualization and design of the study. MZ led the statistical analysis with assistance from ZZ. All authors participated in interpreting the results of the analyses. The manuscript was drafted by MZ and PS. All authors have read and approved the final manuscript.

## Appendix: Simulation experiments

We repeatedly conduct 10 simulation experiments. In each experiment, we first generate a training sample of 1250 observations, each with two predictors (*x*_1_, *x*_2_) and a binary outcome *y*. The first 1000 samples belong to one class (*y *= 0) and the remaining 250 belong to the other (*y *= 1). The two predictors are generated independently using probability distributions specified in Table 8. Then, an independent test sample of 1250 observations are generated using exactly the same mechanism.

**Table 8:** Simulation mechanism. Ratios greater than 1 are bolded.

*j*	Pr(*x*_1_|*y *= 1)	Pr(*x*_1_|*y *= 0)	rx1j MathType@MTEF@5@5@+=feaagaart1ev2aaatCvAUfKttLearuWrP9MDH5MBPbIqV92AaeXatLxBI9gBaebbnrfifHhDYfgasaacPC6xNi=xH8viVGI8Gi=hEeeu0xXdbba9frFj0xb9qqpG0dXdb9aspeI8k8fiI+fsY=rqGqVepae9pg0db9vqaiVgFr0xfr=xfr=xc9adbaqaaeGacaGaaiaabeqaaeqabiWaaaGcbaGaemOCai3aa0baaSqaaiabdIha4naaBaaameaacqaIXaqmaeqaaaWcbaGaemOAaOgaaaaa@316B@	Pr(*x*_2_|*y *= 1)	Pr(*x*_2_|*y *= 0)	rx2j MathType@MTEF@5@5@+=feaagaart1ev2aaatCvAUfKttLearuWrP9MDH5MBPbIqV92AaeXatLxBI9gBaebbnrfifHhDYfgasaacPC6xNi=xH8viVGI8Gi=hEeeu0xXdbba9frFj0xb9qqpG0dXdb9aspeI8k8fiI+fsY=rqGqVepae9pg0db9vqaiVgFr0xfr=xfr=xc9adbaqaaeGacaGaaiaabeqaaeqabiWaaaGcbaGaemOCai3aa0baaSqaaiabdIha4naaBaaameaacqaIYaGmaeqaaaWcbaGaemOAaOgaaaaa@316D@
0	0.40	0.10	**4.00**	0.20	0.10	**2.00**
1	0.40	0.10	**4.00**	0.10	0.15	0.67
2	0.04	0.16	0.25	0.09	0.15	0.60
3	0.03	0.16	0.19	0.10	0.15	0.67
4	0.05	0.16	0.31	0.11	0.15	0.73
5	0.05	0.16	0.31	0.10	0.15	0.67
6	0.03	0.16	0.19	0.10	0.15	0.67

The predictor *x*_1 _is designed to mimic the behavior of h2j, whereas the predictor *x*_2 _is designed to mimic the behavior of a typical h2* covariate such as h2i. In particular, for *x*_1_, we have rx10=rx11
 MathType@MTEF@5@5@+=feaagaart1ev2aaatCvAUfKttLearuWrP9MDH5MBPbIqV92AaeXatLxBI9gBaebbnrfifHhDYfgasaacPC6xNi=xH8viVGI8Gi=hEeeu0xXdbba9frFj0xb9qqpG0dXdb9aspeI8k8fiI+fsY=rqGqVepae9pg0db9vqaiVgFr0xfr=xfr=xc9adbaqaaeGacaGaaiaabeqaaeqabiWaaaGcbaGaemOCai3aa0baaSqaaiabdIha4naaBaaameaacqaIXaqmaeqaaaWcbaGaeGimaadaaOGaeyypa0JaemOCai3aa0baaSqaaiabdIha4naaBaaameaacqaIXaqmaeqaaaWcbaGaeGymaedaaaaa@3737@ = 4 > 1 and rx1j
 MathType@MTEF@5@5@+=feaagaart1ev2aaatCvAUfKttLearuWrP9MDH5MBPbIqV92AaeXatLxBI9gBaebbnrfifHhDYfgasaacPC6xNi=xH8viVGI8Gi=hEeeu0xXdbba9frFj0xb9qqpG0dXdb9aspeI8k8fiI+fsY=rqGqVepae9pg0db9vqaiVgFr0xfr=xfr=xc9adbaqaaeGacaGaaiaabeqaaeqabiWaaaGcbaGaemOCai3aa0baaSqaaiabdIha4naaBaaameaacqaIXaqmaeqaaaWcbaGaemOAaOgaaaaa@316B@ < 1 for all *j *= 2, 3, 4, 5, 6. That is, just like h2j, the recoded scale is optimal for *x*_1_. For *x*_2_, we have rx20
 MathType@MTEF@5@5@+=feaagaart1ev2aaatCvAUfKttLearuWrP9MDH5MBPbIqV92AaeXatLxBI9gBaebbnrfifHhDYfgasaacPC6xNi=xH8viVGI8Gi=hEeeu0xXdbba9frFj0xb9qqpG0dXdb9aspeI8k8fiI+fsY=rqGqVepae9pg0db9vqaiVgFr0xfr=xfr=xc9adbaqaaeGacaGaaiaabeqaaeqabiWaaaGcbaGaemOCai3aa0baaSqaaiabdIha4naaBaaameaacqaIYaGmaeqaaaWcbaGaeGimaadaaaaa@30FE@ = 2 > 1 and rx2j
 MathType@MTEF@5@5@+=feaagaart1ev2aaatCvAUfKttLearuWrP9MDH5MBPbIqV92AaeXatLxBI9gBaebbnrfifHhDYfgasaacPC6xNi=xH8viVGI8Gi=hEeeu0xXdbba9frFj0xb9qqpG0dXdb9aspeI8k8fiI+fsY=rqGqVepae9pg0db9vqaiVgFr0xfr=xfr=xc9adbaqaaeGacaGaaiaabeqaaeqabiWaaaGcbaGaemOCai3aa0baaSqaaiabdIha4naaBaaameaacqaIYaGmaeqaaaWcbaGaemOAaOgaaaaa@316D@ < 1 for all *j *= 1, 2, 3, 4, 5, 6. That is, just like most of the h2* covariates, the recoded scale is close to being optimal for *x*_2_, but it would have been better to separate 0 from 1–6 rather than grouping 0 and 1 together (Table 8). The correct decision is to predict y^
 MathType@MTEF@5@5@+=feaagaart1ev2aaatCvAUfKttLearuWrP9MDH5MBPbIqV92AaeXatLxBI9gBaebbnrfifHhDYfgasaacPC6xNi=xH8viVGI8Gi=hEeeu0xXdbba9frFj0xb9qqpG0dXdb9aspeI8k8fiI+fsY=rqGqVepae9pg0db9vqaiVgFr0xfr=xfr=xc9adbaqaaeGacaGaaiaabeqaaeqabiWaaaGcbaGafmyEaKNbaKaaaaa@2D5E@ = 1 if and only if (*x*_1_, *x*_2_) = (0, 0) or (1, 0).

**Figure 2:**
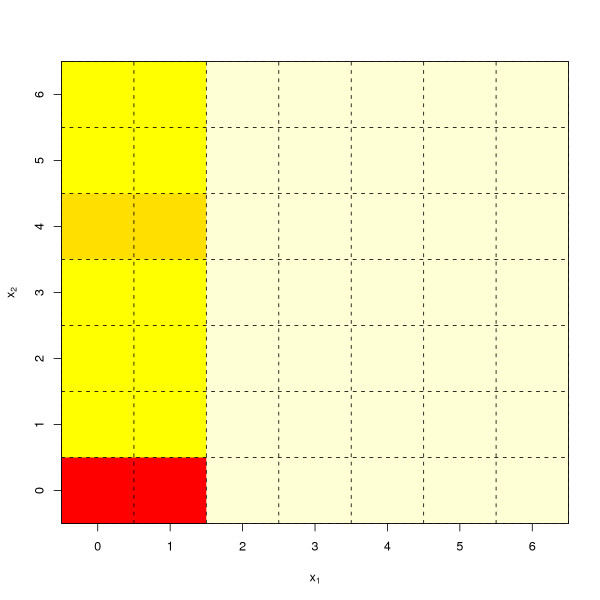
True decision surface for simulation experiments.

The true decision surface is shown in Figure 2. The distributions of (*x*_1_, *x*_2_) are deliberately made somewhat noisy and irregular for *x*_1 _≥ 2 and *x*_2 _≥ 1. As a result, we can see that there is a small but noticeable bump in the true decision surface around (*x*_1_, *x*_2_) = (0, 4) and (1, 4) (Figure 2). This will increase the chance for data-driven algorithms to make mistakes in this region.

We then fit a KNN and an SVM model on the training sample and use them to predict the test sample. We do this once with the predictors (*x*_1_, *x*_2_) in their original scale and once with the predictors recoded according to Table 1 as if they were h2j and h2i.

**Figure 3:**
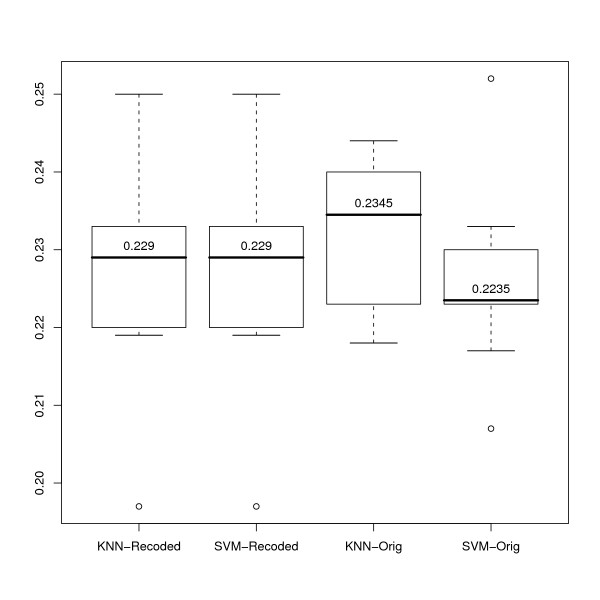
Overall error rates from 10 simulation experiments. KNN and SVM perform comparably with recoded data. KNN performs slightly worse whereas SVM performs slightly better with original data.

The overall error rates of KNN and SVM from the 10 simulations are shown using boxplots in Figure 3. The performances of KNN and SVM are almost identical when applied to the recoded variables, but when applied to the original variables, SVM performs slightly better whereas KNN performs slightly worse.

**Figure 4:**
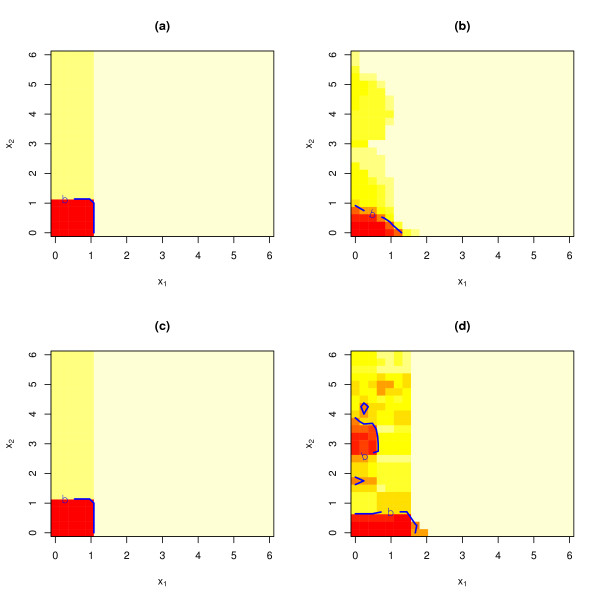
Estimated decision surfaces (averaged over 10 simulations). The contour line labeled "b" is the effective decision boundary. (a) SVM with recoded inputs. (b) SVM with original inputs. (c) KNN with recoded inputs. (d) KNN with original inputs.

Figure 4 gives more insight into why this is the case. Decision surfaces estimated by KNN and SVM from the training samples (averaged over 10 simulations) are displayed. Here, we can see that, when the original scales are used, KNN produces a considerably noisier decision surface whereas SVM is capable of producing a much smoother decision surface. It is also clear that, when the original scales are used, KNN is more likely than SVM to be "fooled" by the extra bump near (*x*_1_, *x*_2_) = (0, 4). In addition, when the original scales are used, SVM can be seen to have a much better chance of making the correct prediction of y^
 MathType@MTEF@5@5@+=feaagaart1ev2aaatCvAUfKttLearuWrP9MDH5MBPbIqV92AaeXatLxBI9gBaebbnrfifHhDYfgasaacPC6xNi=xH8viVGI8Gi=hEeeu0xXdbba9frFj0xb9qqpG0dXdb9aspeI8k8fiI+fsY=rqGqVepae9pg0db9vqaiVgFr0xfr=xfr=xc9adbaqaaeGacaGaaiaabeqaaeqabiWaaaGcbaGafmyEaKNbaKaaaaa@2D5E@ = 0 at (*x*_1_, *x*_2_) = (1, 1).

Finally, it is worth noting that there is considerable noise in our simulated data. Even if we used the true underlying decision surface (Figure 2) to make predictions, we would still make considerable misclassification error. Therefore, a high error rate alone should not be taken as an indication that algorithms such as KNN and SVM are performing poorly. In this case, it is rather an indication that the underlying data are very noisy. In fact, the decision surface estimated by SVM (Figure 4) in this simulation was not far from the true decision surface (Figure 2).

## Pre-publication history

The pre-publication history for this paper can be accessed here:


